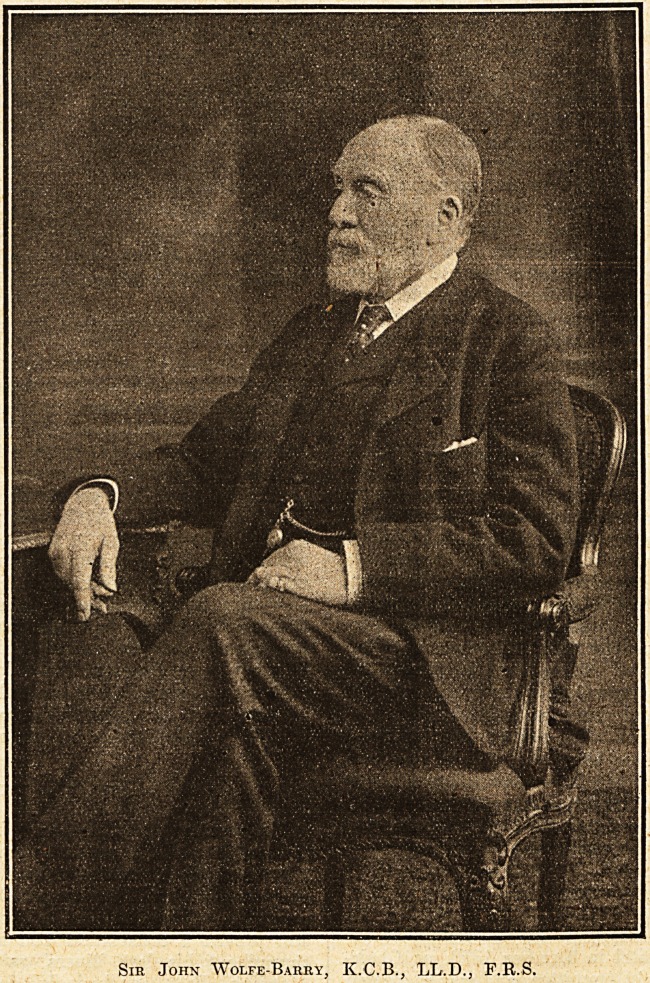# The Late Sir J. Wolfe-Barry, K.C.B.

**Published:** 1918-02-02

**Authors:** 


### THE LATE SIR J. WOLFE-BARRY, K.C.B.

### An Interesting Man and a Lovable Personality.

Sir John Wolfe-Barry, the late chairman of Westminster Hospital, who died last week at the age of eighty-one, was a remarkable member of a remarkable family. When we look at the associations which his family had with London during the nineteenth century, it seems peculiarly fitting that he should have been closely connected with that hospital whose bare name distinguishes it as the hospital in the very heart of ancient London. Westminster seems the inner London of the metropolis, and its material development is naturally mingled with the life-work of London's most prominent engineer. To the stranger two of the most conspicuous architectural points in London are the Houses of Parliament and the Tower Bridge. Sir Charles Barry, the father, built the former, and his son was the engineer of the latter. Both have been severely criticised, but time and London's indifference have made the Houses of Parliament acceptable, and the same acceptance may, less surely, be extended to the bridge. Unlike the Houses of Parliament, which stand alone and dominate the river, the Tower Bridge has its rival in the Tower. Beside that, it is more remarkable as an engineering feat than as incongruous as a piece of modern "Gothic." The Barrys, however, will be permanently associated with the river, for as the father gave London the "Terrace," the son was busy building bridges across the Thames.

Sir John Wolfe-Barry, K.C.B., LL.D., F.R.S.

### A Builder of Bridges.

As one of Sir John Hawkshaw's pupils, bridges and railway engineering interested him from the start, and it was as his pupil that Wolfe-Barry worked on the Cannon Street and Charing Cross railway stations, and consequently on the bridges which carry their traffic over the Thames. Docks and railways in all parts of the world were constructed with his advice as consulting engineer, and to his list of London bridges we must add the Blackfriars railway bridge and the later bridge at Kew. The varied aspects of London's traffic always interested him, and he favoured drastic changes, but he would probably have admitted that little has been done in the direction to which he pointed either before or since the Royal Commission on the subject in 1903, of which he was a member. Of his work on standardisation in engineering, and for the co-ordination of the profession itself, this is not the place to speak.

### The Westminster Hospital's Site.

Interested as he was in London's traffic, and consequently in London sites, it is a pity that he did not see the solution of the vexed question of the site of Westminster Hospital. This site has an institutional and public interest. The hospital has long considered moving. The site which it occupies is of far greater importance than the

### THE LATE SIR J. WOLFE-BARRY, K.C.B.—(*continued*).

building which stands upon it. The hospital authorities have nearly completed the sale on more than one occasion, notably to the Canadian Government in 1914, but some hitch has invariably occurred. Undoubtedly the proper course would be to remove the hospital. This course Sir John Wolfe-Barry approved, and spent much valuable time during his later years in bringing it to fruition. In 1915 he addressed a memorandum on the subject to the President, Vice-Presidents, Governors and subscribers to Westminster Hospital, in which he intimated the removal and rebuilding of Westminster Hospital, which must in all probability be postponed for a considerable time. He deemed it, however, to be desirable (1) to put on record some of the many cogent reasons which led the Governors, after long consideration, to adopt the policy of removal, and (2) to report upon the initiatory steps which had been begun in that direction. His memorandum was made for the purpose of future reference when the scheme again becomes practicable, and constitutes a document of considerable interest, sober and convincing facts and many points, including a description of some of the salient features of the proposed new buildings of the new Westminster Hospital, Clapham Common, with the preliminary plans prepared by Mr. H. Percy Adams, F.R.I.B.A.

### Salvation for a Poor District.

Westminster Hospital at present is no longer in the centre of a poor district, and Sir John Wolfe-Barry gave convincing figures and facts on which he rested his conviction that it was essential to remove it to the selected site at Clapham Common, which is adjacent to a thickly populated and growing neighbourhood of small houses. Here the usefulness of Westminster Hospital must be enormously increased, whilst its removal cannot fail greatly to stimulate and benefit the interests of the Medical School, and prove of hygienic advantage not only to the patients but also to the nurses and staff, owing to their being housed in new and spacious buildings with all modern improvements, situated in a remarkably healthy position, suitable for the best open-air treatment of patients, and providing ample space for recreative purposes.

### Twenty Years Hospital Chairman.

Sir John Wolfe-Barry joined the Committee of Westminster Hospital in October 1898, as Chairman of the Hospital Committee, and was re-elected Chairman every year since that date. His chairmanship is made memorable by the installation of electric light throughout the hospital; new and improved accommodation for nurses; the establishment of an Electrical and Röntgen Ray Department; a Clinical Laboratory opened by the late Lord Lister, and of a Bacterio-Therapeutic Laboratory, towards which the Goldsmiths' Company, through Sir John Wolfe-Barry, gave a grant of £500.

### Sir John Wolfe-Barry's Last Wish.

Sir John Wolfe-Barry was taken ill on the 14th December, and died on the 22nd January, 1918. The *Morning Post* of the 19th January contained a letter from him as Chairman of Westminster Hospital, referring to an appeal for £20,000 for his hospital, which is in urgent need of funds. Towards that appeal he had received £10,000, and in thanking the generous donors he urged those who had not yet contributed to send what they could, so that the remaining £10,000 might be promptly forthcoming to tide Westminster Hospital over this difficult time. This dying request of a most popular and efficient Chairman and great engineer, to whose life work Londoners are, and ever will be, greatly indebted, should result in the prompt contribution of the required £10,000, so that the present and immediate future liabilities of this hospital may be provided for. Sir John Wolfe-Barry took the keenest interest in the progress of this appeal, and we are confident that the whole £20,000 will be subscribed quickly.

### An Interesting Personality.

It is welcome to add that during Sir John Wolfe-Barry's chairmanship the invested funds of Westminster Hospital have increased by £32,000. Upwards of £23,000 of this has been given for endowment purposes, a notable fact in view of the change of opinion in regard to endowments, largely due to modern conditions and developments. Personally Sir John Wolfe-Barry, K.C.B., was a most interesting man to meet and be associated with. He had a pleasant manner, and his methods were so thorough and conscientious as to simplify everything he touched. and to win the support of his associates in exact proportion to the height of their intelligence and the extent of their brain power. His house on Chelsea Embankment was full of interest. There could be seen illustrations of great bridges, and amongst them some of the finest artistic reproductions to be met with anywhere.

### Personal Service and its Fruit.

For twenty years he was Chairman of Westminster Hospital, in connection with which he gave a fine example of personal service of a most practical and unselfish kind, an association which brought him much satisfaction in the later years of his life, and proved a consolation and inspiration to him throughout his last illness, as his letter to the *Morning Post* conclusively demonstrates. We, like the many who knew him and were associated with him in his life's work and had the privilege of being amongst his fellow-workers, shall mourn his loss and take encouragement from his example. Those of us who shared this privilege will look forward and upwards, with ever-increasing confidence, for as long as it is our lot to live in this world and to rest our work on the highest standards of which we are capable.

## Figures and Tables

**Figure f1:**